# Radiolytic Synthesis of Chitosan-Stabilized Silver Nanoparticles via Electron Beam Irradiation for Enhanced Antibacterial Activity Against *Staphylococcus aureus* and *Escherichia coli*

**DOI:** 10.3390/ijms27062569

**Published:** 2026-03-11

**Authors:** Suphalak Khamruang Marshall, Wuttipat Wattanaphonpinich

**Affiliations:** Department of Radiology, Faculty of Medicine, Prince of Songkla University, Songkhla 90110, Thailand

**Keywords:** *Staphylococcus aureus*, *Escherichia coli*, sepsis, silver nanoparticles, antibacterial, chitosan sliver nanoparticles, antimicrobial resistance, electron beam, electron beam irradiation

## Abstract

Antimicrobial resistance is a major global health threat, creating an urgent need for effective non-antibiotic antimicrobial strategies. In this study, CS–AgNPs were synthesized by electron-beam radiolysis, providing a clean, dose-controllable route that avoids additional chemical reducing agents. The effects of irradiation dose and chitosan concentration on nanoparticle formation, physicochemical properties, and antibacterial activity were systematically evaluated. Spectroscopic and structural analyses confirmed the formation of highly crystalline, face-centered cubic silver nanoparticles uniformly dispersed within the chitosan matrix, with Ag–polymer coordination involving –NH_2_ and –OH functional groups. Under the optimal conditions (8 kGy, 0.06 mmol AgNO_3_, and 0.05% *w*/*v* chitosan), ultrasmall, well-dispersed CS–AgNPs were obtained, with an average size of 5.30 ± 2.01 nm and high phase purity. Antibacterial evaluation demonstrated potent, concentration-dependent activity against both Gram-positive *Staphylococcus aureus* and Gram-negative *Escherichia coli*, with low minimum inhibitory and minimum bactericidal concentrations (MIC/MBC = 1.96 µg/mL). These findings define a clear structure–property–activity relationship and support a synergistic antibacterial effect between nanosilver and chitosan, while maintaining favorable in vitro cytocompatibility and hemocompatibility within the effective concentration range. Overall, electron-beam radiolysis represents a promising scalable platform for producing broad-spectrum antimicrobial nanomaterials with potential utility in addressing antimicrobial resistance.

## 1. Introduction

Bacterial infections remain a major global public health challenge, contributing significantly to morbidity and mortality across diverse clinical settings. Five pathogens, *Staphylococcus aureus* (*S. aureus*), *Escherichia coli* (*E. coli*), *Streptococcus pneumonia* (*S. pneumonia*), *Klebsiella pneumonia* (*K. pneumonia*), and *Pseudomonas aeruginosa* (*P. aeruginosa*), cause millions of deaths worldwide annually. According to a study by Gray et al. in 2021, estimated that there were 166 million (95% uncertainty interval 135–201) sepsis cases in 2021 and 21.4 million sepsis-related deaths globally, representing 31.5% of total global deaths [1]. With many survivors having to deal with the repercussions of sepsis for the rest of their lives [2,3]. However, there is increasing interest in the unique physicochemical features of silver nanoparticles (AgNPs), especially their antibacterial, anti-inflammatory, and possible anticancer effects, which have attracted great interest in their potential medicinal use [4,5,6]. AgNPs have a broad spectrum of antibacterial action and are among their most recognized applications. Moreover, AgNPs size is a critical factor in their biological effectiveness, toxicity, and functional performance. The efficacy of the nanoparticles is greatly affected by their size as smaller particles interact more with bacterial membranes by having a larger surface area-to-volume ratio. Studies suggest that AgNPs in the size range of 1–10 nm exhibit the highest antibacterial efficacy due to their ability to penetrate the cell walls of bacteria, generating reactive oxygen species (ROS) and contributing to cell damage and death [7]. AgNPs of 5–20 nm has been reported to effectively inhibit bacterial growth, with smaller particles (5–10 nm) being more effective against Gram-negative bacteria due to their thinner peptidoglycan layer [8].

AgNPs exhibit broad-spectrum antibacterial activity, yet their biomedical application is constrained by aggregation, uncontrolled silver ion release, and potential cytotoxicity. Polymeric stabilization is therefore essential to modulate physicochemical behavior and biological response. Chitosan, a cationic, biocompatible polysaccharide, was selected as a stabilizing matrix due to its amino- and hydroxyl-rich structure, which enables coordination with silver species and enhances colloidal stability. Protonated amino groups further promote electrostatic interaction with negatively charged bacterial membranes, potentially improving antimicrobial efficacy while regulating ion release kinetics [9,10,11]. Synthesis methodology critically governs nanoparticle structure and functionality. Electron beam irradiation offers a clean, initiator-free route to AgNP formation via radiolytic generation of solvated electrons and hydrogen radicals, enabling rapid Ag^+^ reduction without chemical reducing agents. This approach provides precise dose control, minimizes residual contaminants, and enhances reproducibility within polymer matrices [12,13,14]. The integration of chitosan stabilization with electron beam radiolysis thus represents a rational strategy for generating structurally controlled, contamination-free, and biologically functional AgNP systems.

Furthermore, to enhance antibacterial properties, the coating of AgNPs on to surfaces is a strategy that is well-established. Therefore, the objective of this study is to determine the optimal antibacterial performance of chitosan-stabilized silver nanoparticles (CS–AgNPs) synthesized via electron beam irradiation against *S. aureus* ATCC 25923 (Gram-positive) and *E. coli* ATCC 25922 (Gram-negative). *S. aureus* and *E. coli* are known to cause hard-to-treat infections. This highlights the urgent need for new antimicrobials with multi-target actions to reduce resistance. Antimicrobial resistance is increasingly a global health threat. Notably, this study uniquely combines electron beam radiolysis with chitosan stabilization. This approach enables dose-optimized synthesis of AgNPs, with the ability to link controlled physicochemical tuning with exceptionally potent, broad-spectrum antibacterial activity.

**Figure 1:**
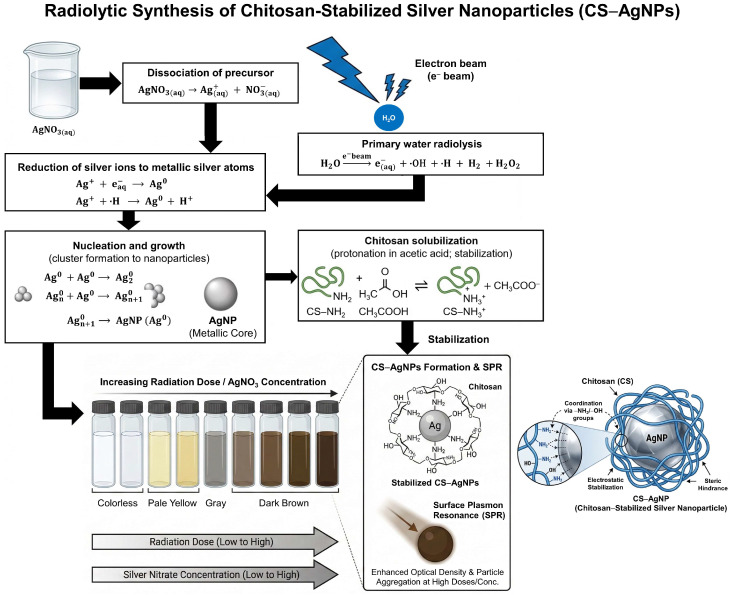
Schematic representation of the radiolytic synthesis of CS–AgNPs via electron beam irradiation. Chitosan and AgNO_3_ (Ag^+^) solutions were exposed to increasing irradiation doses, leading to a progressive color change from colorless to pale yellow, gray, and dark brown, corresponding to nanoparticle nucleation and growth. The color transition reflects the excitation of surface plasmon resonance (SPR) as metallic AgNPs form and become stabilized by chitosan through interactions with amino (–NH_2_) and hydroxyl (–OH) functional groups. Higher radiation doses and AgNO_3_ concentrations promote greater nanoparticle formation and increased optical density, indicating enhanced reduction efficiency and particle aggregation tendency.

Accordingly, this unique study aims to systematically evaluate the influence of irradiation dose and chitosan concentration on the electron beam radiolytic synthesis of CS–AgNPs. The key objective is to control nanoparticle size, dispersion, and physicochemical characteristics relevant to antibacterial performance (Figure 1). By establishing a structure–property relationship, this work seeks to provide a rational, dose-optimized nanomaterial design framework for broad-spectrum antibacterial applications against both Gram-positive *S. aureus* and Gram-negative *E. coli*, thereby supporting the development of alternative antimicrobial strategies in the context of escalating antimicrobial resistance.

## 2. Results

### 2.1. Characterization of CS–AgNPs Synthesized via Electron Beam Irradiation

#### 2.1.1. Physical Characterization and Colorimetric Responses of CS–AgNPs

The synthesis of CS–AgNPs via electron beam irradiation induced distinct visual, chemical, and physical changes, which were closely associated with nanoparticle formation and size. These transformations were evaluated by observing color variations, SPR effects, and the influence of precursor concentration and radiation dose.

**Figure 2:**
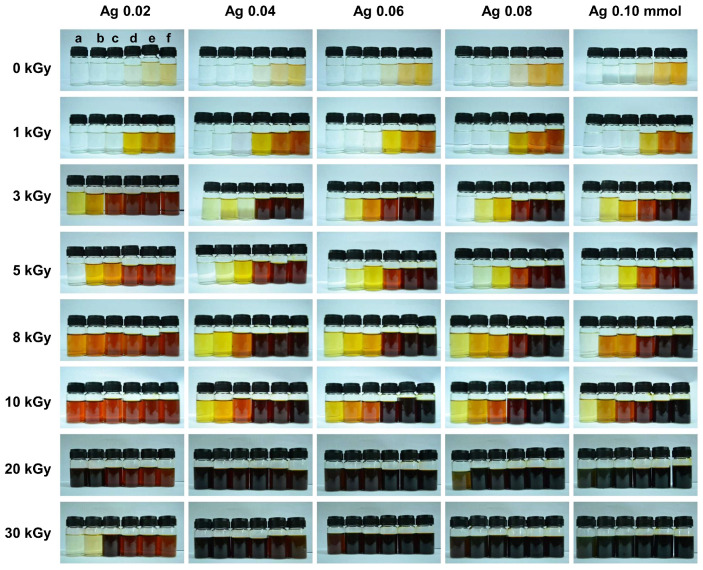
Physical characteristics of chitosan–AgNO_3_ solutions subjected to electron beam irradiation at different doses and precursor concentrations. Samples were prepared with AgNO_3_ concentrations of 0.02, 0.04, 0.06, 0.08, and 0.10 mmol and chitosan concentrations of (**a**) 0.02, (**b**) 0.05, (**c**) 0.10, (**d**) 0.50, (**e**) 1.00, and (**f**) 1.50% (*w*/*v*). The solutions were irradiated at 0, 1, 3, 5, 8, 10, 20, and 30 kGy. The progressive color change from colorless to yellow, brown, and dark brown with increasing radiation dose and precursor concentration indicates the radiolytic formation and growth of CS–AgNPs due to the excitation of SPR phenomena.

Upon electron beam irradiation, the CS–AgNPs solution exhibited a clear and progressive color change from colorless to pale yellow, gray, dark brown, and ultimately black depending on experimental conditions (Figure 2). This visual transformation is indicative of the nucleation and growth of AgNPs and is attributed to SPR, a phenomenon where free electrons on the nanoparticle surface oscillate in resonance with incident light, leading to enhanced absorption and scattering in the visible spectrum. The observed color intensification correlates with increasing nanoparticle concentration and changes in particle size and distribution.

Radiation dose was found to be a critical factor influencing the size and distribution of CS–AgNPs. At lower doses (e.g., <10 kGy), the radiolytic production of reducing species such as hydrated electrons ($e_{aq}^{-}$), hydrogen radicals (·H), and hydroxyl radicals (·OH) was limited, resulting in fewer nucleation events and allowing the formed particles to grow larger. As the dose increased up to 30 kGy, a higher concentration of reducing species accelerated the nucleation rate, leading to the formation of a greater number of smaller nanoparticles. This shift was visibly represented by a darker solution color and sharper SPR absorbance. However, at excessively high doses, secondary effects such as nanoparticle aggregation or chitosan degradation were observed, which can lead to broader size distributions and reduced stability.

Furthermore, it was established that the AgNO_3_ concentration influenced both the quantity and size of nanoparticles formed. At lower concentrations (0.02 mM), the availability of Ag^+^ ions was limited, resulting in fewer nucleation events and relatively small, monodisperse nanoparticles. As the AgNO_3_ concentration increased in size to 0.10 mM, the number of reduced silver atoms rose, promoting greater nanoparticle formation. As a result, this visually led to an enhanced SPR effect and deeper brown coloration. However, without a corresponding increase in chitosan or irradiation energy, higher silver ion concentrations increased the risk of particle aggregation, leading to larger and more polydisperse nanoparticles.

Chitosan served a dual role as a weak reducing agent and an effective capping agent. At low concentrations (e.g., 0.02% *w*/*v*), stabilization was insufficient, allowing particle coalescence and the formation of larger aggregates, resulting in uneven coloration. As chitosan concentration increased to 1.50% *w*/*v*, the solution color intensified from pale yellow to dark brown or black. This color change corresponds to both a more efficient reduction process and enhanced nanoparticle stability. Specifically, the amino (–NH_2_) and hydroxyl (–OH) groups in chitosan coordinated with the CS–AgNPs surface, restricting growth and aggregation, while leading to smaller particle sizes and narrower size distributions. Moreover, the increased viscosity and steric hindrance from the chitosan matrix further contributed to improved colloidal stability.

As a result, the visible color transitions across all tested conditions served as rapid qualitative indicators of underlying chemical reduction and physical nanoparticle formation. Darker colors corresponded with increased nanoparticle concentrations. The smaller average particle sizes were due to higher nucleation rates. Conversely, lighter shades indicated fewer, larger particles or incomplete reduction.

#### 2.1.2. UV–Vis Spectroscopic Analysis of CS–AgNPs

**Figure 3:**
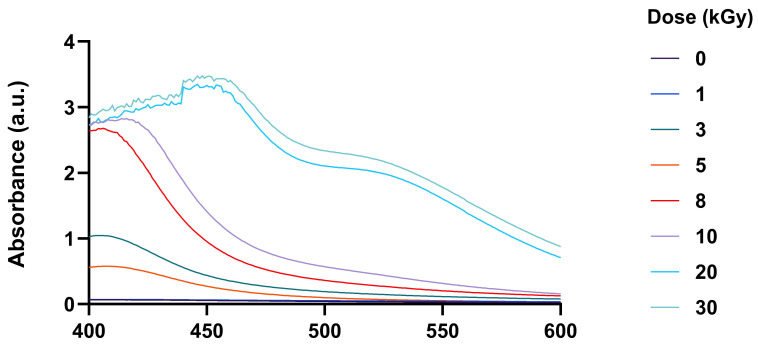
UV–Vis absorption spectra of CS–AgNPs prepared with 0.06 mmol AgNO_3_ and 0.05% (*w*/*v*) chitosan solution and synthesized via electron beam irradiation at doses of 0, 1, 3, 5, 8, 10, 20, and 30 kGy.

The formation of CS–AgNPs via electron beam irradiation was confirmed by UV–Vis spectroscopy. As shown in Figure 3, the UV–Vis spectra of CS–AgNPs irradiated at 3, 5, 8, 10, 20, and 30 kGy exhibited characteristic SPR peaks in the 400–470 nm range. In contrast, the SPR peak was observed in the non-irradiated solution (0 kGy). At 3 kGy, a weak SPR peak emerged, reflecting initial nucleation, whereas irradiation at 8 kGy produced the most intense and narrow peak, indicative of a high concentration of uniformly sized nanoparticles. Higher doses (10–30 kGy) caused peak broadening and slight redshifts, consistent with increased particle size and aggregation.

Additional experiments varying AgNO_3_ (0.02–0.10 mmol) and chitosan concentrations (0.02–1.50% *w*/*v*) demonstrated that nanoparticle size and stability were strongly influenced by solution composition. Moderate chitosan concentrations (0.05–0.10% *w*/*v*) effectively stabilized AgNPs, whereas low or excessively high concentrations led to aggregation or hindered nucleation. Increasing AgNO_3_ concentration enhanced the SPR peak intensity, reflecting higher nanoparticle yield (Figures S1–S5).

As a result, electron beam irradiation provides a controlled and effective method for synthesis of CS–AgNP. Irradiation at 8 kGy of a solution containing 0.05% (*w*/*v*) chitosan and 0.06 mmol AgNO_3_ yielded the smallest and most uniform nanoparticles. These results emphasize the critical roles of irradiation dose and chitosan-mediated stabilization in optimizing nanoparticle formation via radiolytic reduction.

#### 2.1.3. Morphology Analysis and Particle Size Distribution Using Transmission Electron Microscopy (TEM)

**Figure 4:**
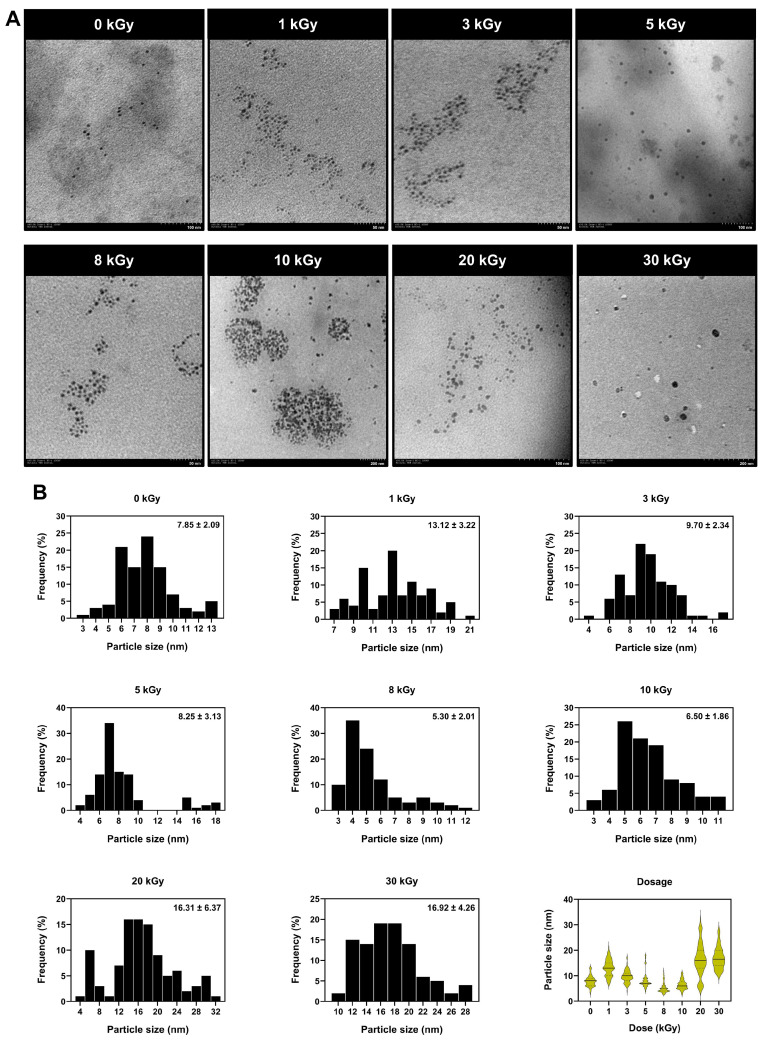
TEM images and particle size distribution of CS–AgNPs synthesized via electron beam irradiation at various doses. (**A**) TEM micrographs of CS–AgNPs prepared using 0.06 mmol AgNO_3_ and 0.05% (*w*/*v*) chitosan solution, irradiated at 0, 1, 3, 5, 8, 10, 20, and 30 kGy. (**B**) Particle size distribution of CS–AgNPs. Histograms represent the frequency distribution of particle sizes measured from TEM micrographs, while the violin plot summarizes the overall variation in particle size as a function of irradiation dose.

The characteristics and sizes of AgNPs synthesized by radiolysis with electron beam irradiation vary depending on the concentration of AgNO_3_ solution, the concentration of chitosan, and the applied level of electron beam dose. Most of the particles exhibit a spherical shape with an average diameter ranging from 3 to 25 nm, as shown in Figure 4. Upon examining the formation of AgNPs, it is evident that chitosan plays a significant role in preventing the aggregation of very small particles and effectively stabilizing the AgNPs.

From the analysis using TEM, the study results show that AgNPs are spherical in shape and decrease in size when exposed to electron beam acceleration in the range of 0–30 kGy. The particle sizes corresponding to electron beam accelerations of 0, 1, 3, 5, 8, 10, 20, and 30 kGy were 9, 11, 9, 8, 5, 6, 14, and 16 nm, respectively. When considered alongside the light absorption values, using 0.05% (*w*/*v*) chitosan and 0.06 mM AgNO_3_ solution, it was found that the measured particle sizes correlated with the absorption values. Specifically, the absorption increased with increasing radiation dose over the range 0–10 kGy, indicating an increase in surface area or a decrease in particle size. However, at higher doses of 20 and 30 kGy, the particle size increased slightly. CS–AgNPs synthesized at 8 kGy exhibited the smallest average particle size (~5.30 ± 2.01 nm) with a narrow size distribution, whereas samples irradiated at 30 kGy displayed larger particles (~10–20 nm) and broader distributions. These results indicate that 8 kGy is the optimal irradiation dose for generating small, monodisperse nanoparticles.

#### 2.1.4. Hydrodynamic Size Distribution and Surface Charge Characterization

Dynamic light scattering (DLS) analysis revealed distinct colloidal behaviors among the tested systems (Table 1). AgNO_3_ showed no defined particle size distribution, confirming its presence as dissolved ionic species rather than nanoparticles. The low zeta potential (−8 ± 2 mV) further indicates the absence of colloidal stabilization. The CS–HOAc solution exhibited a broad hydrodynamic diameter of 220 ± 30 nm with a polydispersity index (PDI) of 0.45, consistent with polymer chain aggregation. The high positive zeta potential (42 ± 3 mV) reflects protonated amino groups (–NH_3_^+^), which confer strong electrostatic stabilization. In contrast, CS–AgNPs exhibited a significantly smaller hydrodynamic diameter of 55 ± 6 nm and a lower PDI, indicating moderately monodisperse nanoparticle formation. The reduced size relative to CS–HOAc suggests polymer compaction around the metallic core during radiolytic synthesis. The positive zeta potential (34 ± 2 mV) confirms effective chitosan surface coverage and electrostatic stability, with the slight decrease compared to CS–HOAc likely resulting from silver coordination effects.

Collectively, these findings confirm successful formation of chitosan-stabilized AgNPs with improved colloidal stability compared to precursor systems. The maintained positive surface charge further supports enhanced interaction potential with negatively charged bacterial membranes, consistent with the observed antibacterial and antibiofilm activities.

### 2.2. Analysis of Morphology and Crystallinity of CS–AgNPs by X-Ray Diffractometer (XRD)

**Figure 5:**
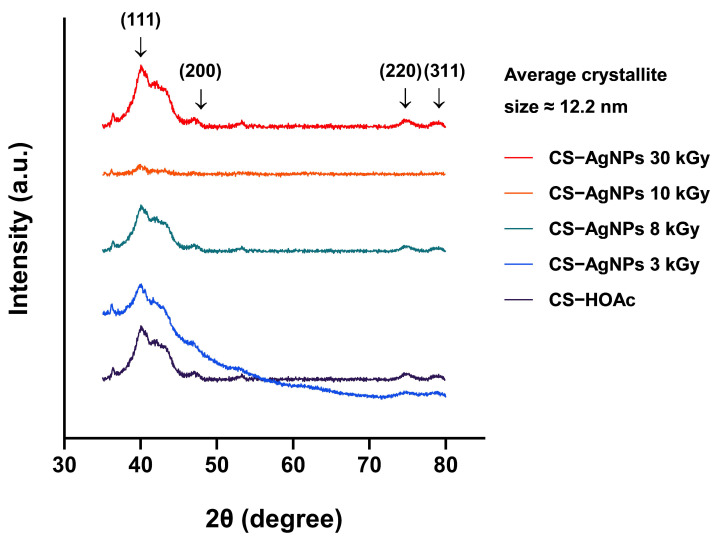
XRD patterns of CS–HOAc and CS–AgNP solutions synthesized by electron beam irradiation at doses of 3, 8, 10, and 30 kGy, using 0.06 mmol AgNO_3_ and 0.05% (*w*/*v*) chitosan.

Figure 5 shows the XRD patterns of chitosan–silver solutions irradiated at 0–30 kGy (0.05% *w*/*v* chitosan; 0.06 mmol AgNO_3_). The non-irradiated sample exhibited no characteristic silver reflections, confirming the absence of crystalline Ag formation and the predominance of an amorphous chitosan matrix.

Upon electron beam irradiation, distinct diffraction peaks appeared at 2θ = 40.2°, 48.9°, 75.5°, and 78.2°, corresponding to the (111), (200), (220), and (311) planes of face-centered cubic (FCC) Ag (JCPDS 04-0783). These reflections confirm radiolytic reduction of Ag^+^ to crystalline Ag^0^ within the polymer matrix. The (111) peak was dominant across irradiated samples, indicating preferential growth along the thermodynamically stable close-packed plane of FCC silver. Progressive peak sharpening and intensity enhancement with increasing dose (3–30 kGy) indicate dose-dependent nucleation and crystal growth, consistent with increased production of solvated electrons ($e_{aq}^{-}$) and hydrogen radicals (H·) that accelerate Ag^+^ reduction kinetics.

Crystallite size (D) was estimated from the full width at half maximum (FWHM) of the (111) reflection using the Scherrer equation. The calculated average crystallite size was approximately 12.2 nm at 30 kGy, indicating nanoscale crystalline domain formation. The reduction in peak broadening with increasing dose suggests enhanced crystallinity and increased crystallite growth, consistent with higher radical flux promoting sustained Ag^0^ nucleation and coalescence. No secondary phases (e.g., Ag_2_O) were detected, confirming phase purity. The broad background halo corresponds to the amorphous chitosan matrix, which remained structurally intact during irradiation.

Collectively, these results demonstrate that electron beam dose governs both nucleation density and crystallite growth kinetics, enabling controlled formation of phase-pure FCC AgNPs with tunable crystalline domain size within the chitosan matrix.

### 2.3. Fourier Transform Infrared Spectrophotometer (FTIR) Analysis

**Figure 6:**
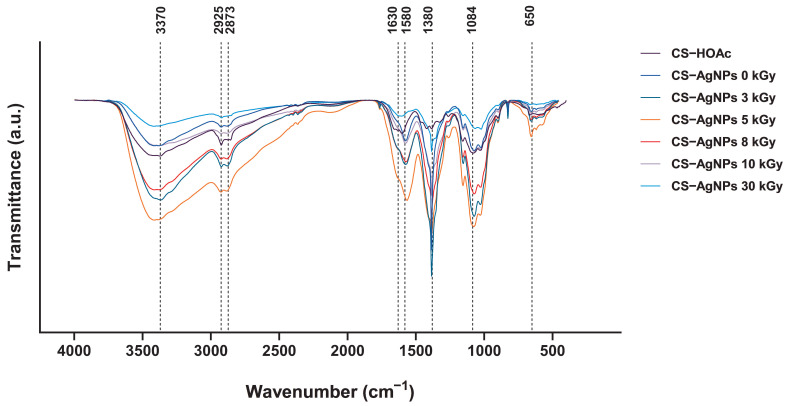
FTIR spectra of CS–HOAc and CS–AgNPs solutions synthesized radiolytically via electron beam irradiation at doses of 0, 3, 5, 8, 10, and 30 kGy, with AgNO3 concentration of 0.06 mmol and chitosan concentration of 0.05% (*w*/*v*).

The FTIR spectra of CS–AgNP systems synthesized via electron beam irradiation at various doses are shown in Figure 6. Characteristic bands were observed at 3370 cm^−1^ (O–H/N–H stretching), 2925 and 2873 cm^−1^ (C–H stretching), 1630 cm^−1^ (amide I, C=O stretching), 1580 cm^−1^ (amide II, N–H bending), 1380 cm^−1^ (C–N stretching), 1084 cm^−1^ (C–O–C/C–O stretching), and 650 cm^−1^ (Ag–O vibration).

Electron beam irradiation induced dose-dependent spectral modifications, indicating Ag^+^ reduction and interfacial interaction between AgNPs and chitosan functional groups. The increased intensity of the amide I band at 1630 cm^−1^ suggests enhanced coordination between carbonyl groups and AgNPs, consistent with Ag–O interactions. Similarly, the intensified amide II band at 1580 cm^−1^ indicates participation of amino (–NH_2_) groups in nanoparticle stabilization, likely through nitrogen lone-pair coordination with metallic silver. Changes in the 1084 cm^−1^ band further support involvement of hydroxyl functionalities in nanoparticle binding. The emergence of a weak band near 650 cm^−1^ is attributed to Ag–O vibrations, supporting the formation of Ag–O–C interfacial linkages within the polymer matrix.

Collectively, these results demonstrate that electron beam irradiation not only reduces Ag^+^ to Ag^0^ but also promotes strong coordination-driven stabilization of AgNPs by amino and hydroxyl groups of chitosan, enhancing nanocomposite integrity and dispersion stability. Such controlled polymer–metal interactions are expected to contribute to sustained antimicrobial functionality while maintaining matrix stability.

### 2.4. Antibacterial Performance of CS–AgNPs Against S. aureus and E. coli

#### 2.4.1. Antibacterial Efficacy of CS–AgNPs Against *S. aureus* and *E. coli* Evaluated by Disc Diffusion Assay

**Figure 7:**
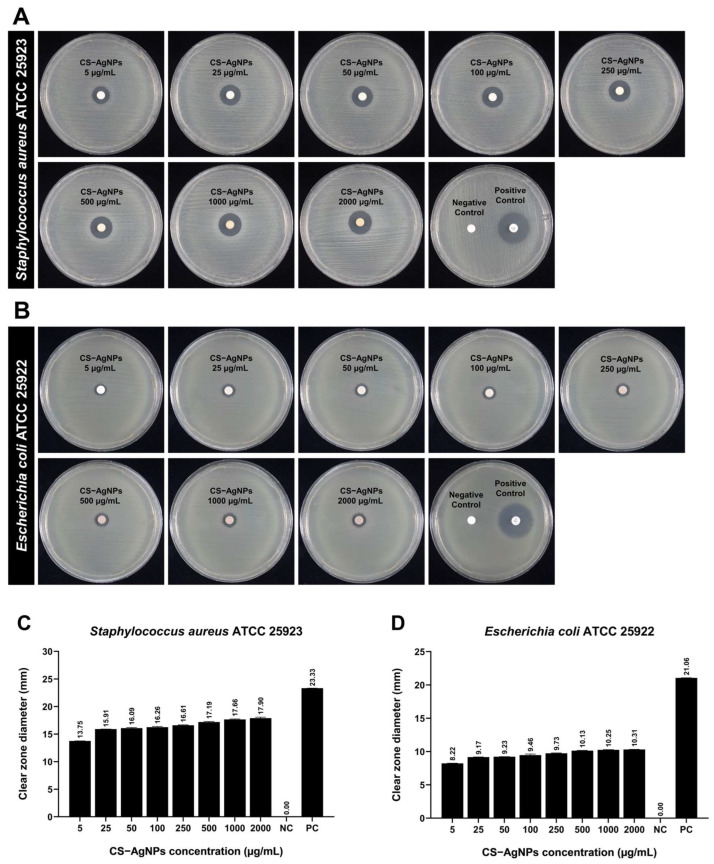
Antibacterial efficacy of CS–AgNPs synthesized via electron beam irradiation against *S. aureus* and *E. coli*. Representative agar disc diffusion plates showing inhibition zones produced by CS–AgNPs at different concentrations (5–2000 µg/mL), compared with the negative control (NC; distilled water) and positive control (PC; Gentamicin) for (**A**) *S. aureus* and (**B**) *E. coli*. Quantitative analysis of inhibition zone diameters (mean ± standard deviation, *n* = 3) demonstrating a concentration-dependent antibacterial activity of CS–AgNPs for (**C**) *S. aureus* and (**D**) *E. coli*.

The antibacterial performance of CS–AgNPs synthesized via electron beam irradiation was evaluated against *S. aureus* and *E. coli* using the disc diffusion method (Figure 7). The inhibition zones formed around each disc demonstrate a concentration-dependent antibacterial response for both bacterial strains. No inhibition was observed in the negative control (distilled water), while the positive control (Gentamicin) produced clear zones of 23.32 ± 0.03 mm for *S. aureus* and 21.06 ± 0.01 mm for *E. coli*, confirming the reliability of the assay. CS–AgNPs exhibited substantial antibacterial activity at low concentrations (5 µg/mL), producing inhibition zones of 13.75 ± 0.02 mm and 8.22 ± 0.03 mm for *S. aureus* and *E. coli*, respectively. As the nanoparticle concentration increased from 5 to 2000 µg/mL, the clear zone diameter gradually increased, reaching 17.90 ± 0.17 mm for *S. aureus* and 10.31 ± 0.01 mm for *E. coli*. This trend indicates a dose-dependent enhancement of antibacterial efficacy, which can be attributed to the greater availability of Ag^+^ ions and increased nanoparticle–cell membrane interactions at higher concentrations.

The difference in susceptibility between the two bacterial strains reflects their distinct cell wall architectures. The Gram-positive *S. aureus* showed higher sensitivity to CS–AgNPs than the Gram-negative *E. coli*. This observation aligns with previous studies, indicating that the thick peptidoglycan layer and absence of an outer lipopolysaccharide membrane in Gram-positive bacteria facilitate greater nanoparticle adhesion and Ag^+^ ion penetration [15,16]. In contrast, the outer membrane of *E. coli* acts as a permeability barrier, limiting Ag^+^ diffusion and nanoparticle binding [17,18]. The antibacterial mechanism of CS–AgNPs likely involves multiple synergistic effects: (i) release of Ag^+^ ions disrupting bacterial enzymatic and respiratory functions; (ii) generation of ROS leading to oxidative damage; and (iii) electrostatic interaction between positively charged chitosan molecules and negatively charged bacterial surfaces, enhancing nanoparticle attachment and membrane permeability [19,20]. The chitosan matrix further stabilizes AgNPs, allowing sustained ion release and prolonged antibacterial activity. Overall, these findings confirm that CS–AgNPs synthesized by electron beam irradiation exhibit broad-spectrum antibacterial efficacy, with enhanced activity against *S. aureus*.

#### 2.4.2. Antibacterial Activity of CS–AgNPs Against *S. aureus* and *E. coli*: MIC and MBC Determination

**Figure 8:**
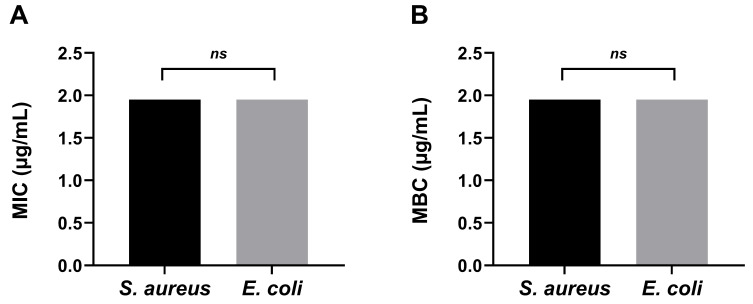
Determination of MIC and MBC of CS–AgNPs against *S. aureus* and *E. coli*. (**A**) MIC values and (**B**) MBC values obtained using the broth microdilution method. Concentrations are expressed as the total mass concentration of the CS–AgNP dispersion (µg/mL) in a well-dispersed nanoparticle suspension. Data are presented as mean ± standard deviation (*n* = 3 independent experiments). Statistical analysis was conducted using one-way ANOVA followed by Tukey’s post hoc test, and pairwise comparisons between bacterial species were performed using an unpaired two-tailed Student’s *t*-test. No statistically significant differences were observed (*ns*, *p* > 0.05).

The minimum inhibitory concentration (MIC) and minimum bactericidal concentration (MBC) of CS–AgNPs against *S. aureus* and *E. coli* are presented in Figure 8. Both bacterial strains exhibited MIC and MBC values of 1.96 µg/mL, reported as the total mass concentration of the CS–AgNP dispersion in a well-dispersed nanoparticle suspension obtained using the broth microdilution method. These findings indicate that CS–AgNPs demonstrate quantifiable antibacterial activity under the experimental in vitro conditions. No statistically significant differences were observed between *S. aureus* and *E. coli* for either MIC or MBC values (*ns*, *p* > 0.05).

Although Gram-positive and Gram-negative bacteria differ structurally in cell envelope composition, silver nanoparticles exert antibacterial effects through multiple concurrent mechanisms, including membrane perturbation, ROS generation, and interference with intracellular biomolecules. These multi-target pathways may reduce dependence on outer membrane architecture and could contribute to the comparable MIC and MBC values observed in this study. The antibacterial response is likely influenced by the combined presence of silver species and chitosan stabilization, where silver contributes to membrane destabilization and oxidative damage, while chitosan enhances nanoparticle dispersion stability and promotes electrostatic interaction with negatively charged bacterial surfaces.

Overall, the findings demonstrate that CS–AgNPs synthesized via electron beam irradiation exhibit measurable in vitro antibacterial activity against both tested strains at the reported concentration range. These results support their potential antimicrobial applicability; however, further studies under physiologically relevant and clinically representative conditions are required to confirm broader translational relevance.

#### 2.4.3. Dose-Dependent Inhibition and Disruption of Bacterial Biofilms

**Figure 9:**
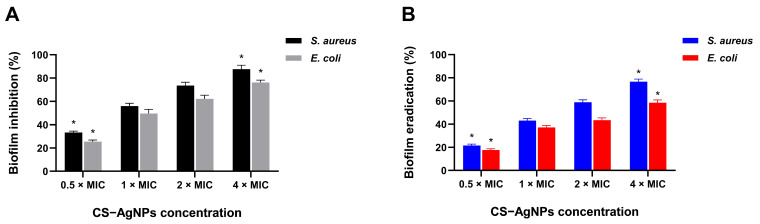
Concentration-dependent inhibition and eradication of bacterial biofilms by CS–AgNPs. (**A**) Inhibition of biofilm formation by *S. aureus* and *E. coli* following 24 h incubation with CS–AgNPs at 0.5 × MIC to 4 × MIC. (**B**) Disruption of pre-formed mature biofilms after 24 h treatment under identical concentration conditions. Biofilm biomass was quantified using crystal violet staining and expressed as percentage reduction relative to untreated controls. Data are presented as mean ± standard deviation (*n* = 3). Statistical significance was determined using one-way ANOVA followed by Tukey’s post hoc test (* *p* < 0.05).

CS–AgNPs demonstrated pronounced, concentration-dependent antibiofilm activity against both *S. aureus* and *E. coli* (Figure 9). In the biofilm inhibition model, treatment at 0.5 × MIC reduced biomass by ~33% and ~26% for *S. aureus* and *E. coli*, respectively. Inhibition increased progressively with concentration, reaching ~88% (*S. aureus*) and ~76% (*E. coli*) at 4 × MIC (*p* < 0.05).

In the mature biofilm model, CS–AgNPs retained substantial disruptive capacity. Eradication efficiencies ranged from ~21–77% for *S. aureus* and ~18–59% for *E. coli* across the tested concentrations, with maximal effects observed at 4 × MIC. Although eradication was consistently lower than inhibition, this pattern aligns with the structural resilience of established extracellular polymeric substance (EPS) matrices.

Notably, *S. aureus* biofilms exhibited slightly greater susceptibility than *E. coli*, potentially reflecting differences in cell envelope architecture and matrix composition. Collectively, these findings indicate that radiolytically synthesized CS–AgNPs effectively interfere with both early biofilm development and established biofilm integrity, reinforcing their therapeutic relevance for biofilm-associated infections.

### 2.5. In Vitro Cytocompatibility and Hemocompatibility Assessment

#### 2.5.1. Assessment of In Vitro Cytocompatibility in HaCaT Keratinocytes

**Figure 10:**
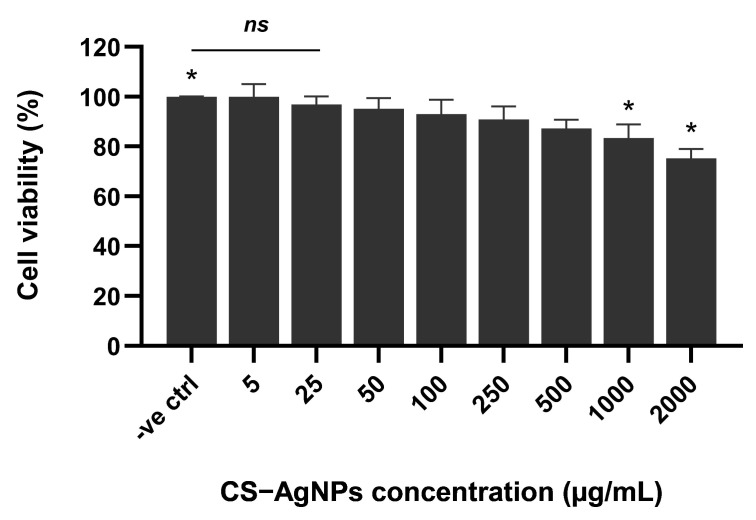
In vitro cytocompatibility of CS–AgNPs toward HaCaT keratinocytes after 48 h of exposure. Cell viability was assessed using the MTT assay at concentrations ranging from 5 to 2000 μg/mL. Data are presented as mean ± standard deviation (*n* = 3). Untreated cells served as the negative control (100% viability). Statistical significance was determined relative to the negative control (*p* < 0.05), while “*ns*” indicates no significant difference. Statistical significance was determined using one-way ANOVA followed by Tukey’s post hoc test (* *p* < 0.05).

The cytocompatibility of CS–AgNPs toward HaCaT keratinocytes was evaluated using the MTT assay after 48 h of exposure at concentrations ranging from 5 to 2000 μg/mL. As shown in Figure 10, cell viability remained above 90% at concentrations up to 500 μg/mL, indicating minimal cytotoxic effects at low to moderate doses. No statistically significant difference (*ns*) was observed between the negative control and concentrations of 5–100 μg/mL. A slight but statistically significant reduction in cell viability was observed at 1000 μg/mL (*p* < 0.05), where viability decreased to approximately 83%. A more pronounced reduction was detected at 2000 μg/mL (*p* < 0.05), with cell viability decreasing to approximately 75%. Importantly, cell viability remained above 80% at concentrations up to 1000 μg/mL, suggesting acceptable cytocompatibility within the antibacterial working concentration range. These findings indicate a concentration-dependent cytotoxic response, with higher nanoparticle concentrations inducing moderate reductions in metabolic activity. Therefore, CS–AgNPs demonstrated in vitro cytocompatibility in HaCaT cells at concentrations relevant for antibacterial applications.

#### 2.5.2. In Vitro Hemolytic Activity and Blood Compatibility Assessment

**Figure 11:**
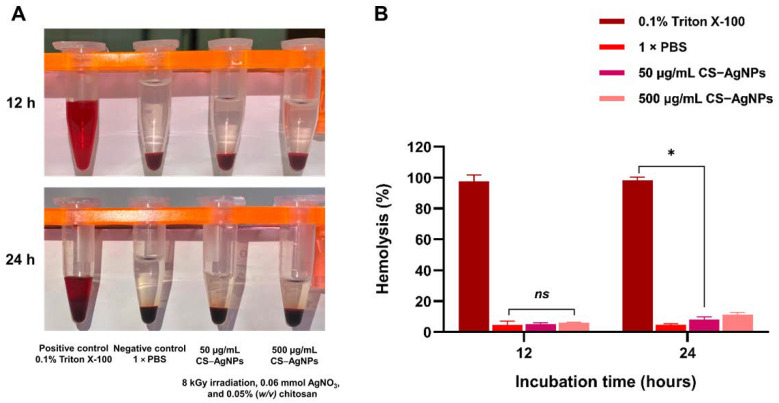
In vitro hemocompatibility assessment of CS–AgNPs. (**A**) Representative images of red blood cell (RBC) suspensions after 12 and 24 h of incubation with 0.1% Triton X-100 (positive control), 1 × PBS (negative control), and CS–AgNPs at 50 and 500 μg/mL (8 kGy irradiation; 0.06 mmol AgNO_3_; 0.05% *w*/*v* chitosan). Visible red coloration in the supernatant indicates hemoglobin release due to membrane disruption. (**B**) Quantitative analysis of hemolysis percentage after 12 and 24 h of incubation. Data are presented as mean ± standard deviation (*n* = 3). Statistical significance was determined relative to the negative control (* *p* < 0.05), while “*ns*” indicates no significant difference.

CS–AgNP-induced hemolytic activity was assessed following 12 and 24 h of incubation with human red blood cells (Figure 11). As anticipated, the positive control (0.1% Triton X-100) produced nearly complete hemolysis (~100%) at both time points, whereas the negative control (1 × PBS) exhibited negligible hemolysis (<5%), confirming the reliability and validity of the assay. After 12 h of exposure, CS–AgNPs at concentrations of 50 and 500 μg/mL induced low levels of hemolysis (approximately 5–7%), with no statistically significant difference compared to the negative control. Following 24 h of incubation, a modest but statistically significant increase in hemolysis was observed at 500 μg/mL (approximately 10–12%), while the 50 μg/mL concentration remained within the non-hemolytic range. Collectively, these findings demonstrate that CS–AgNPs exhibit favorable hemocompatibility at lower concentrations, with only mild, concentration- and time-dependent hemolytic effects detected at higher doses.

## 3. Discussion

Electron beam irradiation enabled controlled synthesis of CS–AgNPs, with nanoparticle formation governed by irradiation dose, AgNO_3_ concentration, and chitosan content. As shown in Figure 2, progressively darker coloration after irradiation is consistent with increased radiolytic reduction of Ag^+^ and greater formation of plasmonic silver nanostructures. This is supported by the UV–Vis spectra in Figure 3, where the characteristic SPR band at 400–470 nm confirms AgNP formation, while changes in band intensity and profile reflect differences in particle size, dispersity, aggregation, and the local dielectric environment. These findings indicate that irradiation conditions modulated the balance between nucleation and particle growth, with chitosan acting as a stabilizing matrix that limited coalescence. Accordingly, chitosan at 0.05–0.10% (*w*/*v*) provided the most effective stabilization, and the formulation containing 0.05% (*w*/*v*) chitosan and 0.06 mmol AgNO_3_ irradiated at 8 kGy produced the smallest and most uniform particles. TEM analysis (Figure 4) further confirmed a dose-dependent effect, with predominantly spherical AgNPs increasing from ~5 nm at 8 kGy to 20–30 nm at 30 kGy, indicating that moderate irradiation favored smaller, more monodisperse particles, whereas higher doses promoted growth-dominant behavior. Under near-neutral conditions, the intrinsic antibacterial contribution of chitosan is likely limited because its activity depends strongly on protonation [9,10,21], suggesting that biological effects in this system are driven mainly by nanosilver, while chitosan contributes primarily to colloidal stabilization. Compared with other radiation-assisted or green synthesis methods [11,12,13,14,22,23], this electron beam approach offers initiator-free reduction, reduced chemical residuals, and tunable control over nanoparticle formation. These observations are consistent with those reported by Huang et al., who demonstrated that by increasing γ-irradiation doses led to smaller, more uniform AgNPs by enhancing radiolytic reduction and stabilizing the chitosan functional groups. This study supports the notion that the strong correlation between particle size and γ-irradiation dose supports this established mechanism, wherein higher doses generate a greater number of reducing species (e.g., hydrated electrons and hydrogen radicals). Resulting in accelerated nucleation and minimizing nanoparticle coalescence [24]. Additionally, Hettiarachchi and Wickramarachchi reported a similar trend in γ-irradiated chitosan–silver systems, in which similarly nanoparticle size decreased up to a critical irradiation dose before aggregation occurred at higher irradiation intensities due to secondary collisions and localized heating effects [25]. Furthermore, the spherical morphology of the nanoparticles observed in this study is consistent with reports by Yoksan and Chirachanchai describing uniform, spherical AgNPs with diameters in the range of 7–30 nm produced via radiation-assisted synthesis routes [26,27]. The nanoparticle spherical geometry is attributed to isotropic growth under uniform radiolytic conditions, while the chitosan amino and hydroxyl functional groups act as stabilizing ligands, which, through electrostatic repulsion and steric hindrance, prevent aggregation. As reported by Twu et al., increasing chitosan concentration enhances steric stabilization, leading to smaller and more monodisperse AgNPs. In the present study, a chitosan concentration of 0.05% (*w*/*v*) provided effective capping without inducing excessive solution viscosity, thereby optimizing nanoparticle dispersion and stability [28]. As a result, the TEM analysis demonstrates that electron beam irradiation enables precise control over the size and morphology of CS–AgNPs, with an optimal dose of 8 kGy yielding small, uniformly distributed, spherical nanoparticles.

XRD analysis confirmed the formation of crystalline metallic silver in the CS–AgNP composites, with distinct diffraction peaks characteristic of face-centered cubic (FCC) silver observed in Figure 5. These reflections matched the standard JCPDS pattern for metallic silver (No. 04-0783), confirming radiolytic reduction of Ag^+^ to Ag^0^ and crystallization of AgNPs within the chitosan matrix. The absence of additional peaks attributable to silver oxide or other secondary phases further supports selective formation of metallic AgNPs. This diffraction pattern is consistent with previous reports of chemically and radiation-synthesized CS–AgNPs, including those of Hegazy and Mahmoud and Ediyilyam et al. [29,30]. This pattern is consistent with previous reports by Ali et al. and Mishra and Kannan in AgNP systems. Such preferential orientation may increase surface reactivity and facilitate Ag^+^ release, which could contribute to enhanced antibacterial activity [31,32]. A dose-dependent increase in crystallinity was evident from the progressive sharpening and increased intensity of the diffraction peaks with increasing electron beam dose. This pattern suggests that higher irradiation promoted more effective nucleation and improved atomic ordering of the silver phase within the chitosan matrix, consistent with previous observations by Patel et al. [33]. This is consistent with previous reports by Hegazy and Mahmoud and Zienkiewicz-Strzałka et al. The broad amorphous background further indicates that the chitosan matrix retained its polymeric character, thereby supporting its function as a stabilizing and capping phase, consistent with the role described by Goy et al. [29,34,35]. Accordingly, the XRD findings confirm that electron beam irradiation is a controllable and clean route for generating highly crystalline, phase-pure AgNPs within a biopolymer matrix.

FTIR analysis confirmed that chitosan retained its key functional groups after electron beam irradiation and that these groups contributed to nanoparticle stabilization (Figure 6). The persistence of the characteristic bands indicates preservation of the chitosan backbone, while increased intensity and slight shifts in the amide region suggest interaction between silver species and the amine/carbonyl groups. These changes support a capping and stabilizing role for chitosan, consistent with previous reports [36,37,38]. A weak band near 650 cm^−1^ appeared in the irradiated samples, consistent with metal–oxygen vibration and suggesting formation of Ag–O–C interactions within the polymer matrix. This low wave number feature supports incorporation of silver into the chitosan network and is consistent with similar CS–AgNP spectra reported by Ramli et al. and Kumar-Krishnan et al. [39,40]. This interpretation is consistent with previous reports describing Ag–O–C coordination as a key contributor to nanocomposite stability and antibacterial performance [41,42].

The disc diffusion assay (Figure 7) showed that CS–AgNPs exerted clear antibacterial activity against both *S. aureus* and *E. coli*, with inhibition zones increasing in a dose-dependent manner. This pattern indicates that higher nanoparticle loading enhanced bactericidal activity, likely through greater Ag^+^ release and increased nanoparticle–membrane interaction. The inhibition zones observed here were comparable to those reported for other chitosan–silver systems, including the values described by Wei et al., indicating that the present electron beam approach achieves similar, and in some cases slightly stronger, antibacterial performance than conventional chemical synthesis [43]. Potara et al. similarly reported synergistic antibacterial activity in chitosan–silver nanocomposites, attributed to electrostatic attraction between the positively charged chitosan matrix and the negatively charged bacterial surface. The same mechanism likely contributed here, particularly against *S. aureus*, where the thick peptidoglycan layer may facilitate nanoparticle adhesion and strengthen growth inhibition [44]. In contrast, *E. coli* consistently exhibits lower susceptibility to AgNPs due to the presence of an outer lipopolysaccharide membrane that limits nanoparticle penetration. Studies by Goy et al. and Chicea et al. reported smaller inhibition zones for *E. coli* compared to *S. aureus*, which is consistent with the present findings [35,45]. El Hassanen et al. likewise identified chitosan–silver nanoparticles as the most effective among several metallic nanoparticle systems, particularly against *S. aureus*. This enhanced activity was attributed to the combined effects of silver-driven membrane damage and ROS generation, together with the stabilizing and biointeractive roles of chitosan [46].

The antibacterial data (Figure 8) indicate that the MIC and MBC values obtained here are at the lower end of, or below, those commonly reported for chitosan–silver nanoparticle systems against *S. aureus* and *E. coli*. This pattern suggests relatively strong antibacterial potency, which is consistent with the smaller particle size, improved dispersion, and favorable surface characteristics of the present CS–AgNPs [47]. Likewise, many chitosan-functionalized AgNP systems show bactericidal activity in the tens of µg/mL range, with differences between *E. coli* and *S. aureus* reflecting the influence of nanoparticle architecture and assay conditions on bacterial susceptibility [48]. Mechanistically, the strong antibacterial activity of CS–AgNPs is consistent with established AgNP effects, including membrane disruption, ROS-mediated oxidative stress, and interference with essential cellular processes, while chitosan likely enhances colloidal stability and bacterial contact through electrostatic interactions. Together, these effects provide a plausible basis for additive or synergistic antibacterial activity [49,50].

The CS–AgNPs showed good cytocompatibility toward HaCaT cells and acceptable hemocompatibility toward human erythrocytes at lower concentrations, with only mild concentration- and time-dependent toxicity at higher exposure. This pattern likely reflects chitosan-mediated colloidal stabilization and moderated Ag^+^ release, which limit cellular and membrane damage while preserving antimicrobial activity. The high viability at antibacterial working concentrations supports this protective effect. The modest hemolysis observed only at elevated doses is likewise consistent with the known concentration-dependent ability of AgNPs to induce oxidative erythrocyte membrane damage and hemoglobin release [51]. Hemolysis remained below 5% at 50 μg/mL and increased only slightly at 500 μg/mL after 24 h, indicating limited erythrocyte membrane disruption at clinically relevant concentrations. Together with the antibacterial data, this suggests a measurable therapeutic window between antimicrobial efficacy and host cell safety. This interpretation is consistent with prior work indicating that dose optimization and surface functionalization are critical for balancing antibacterial potency with biocompatibility [52].

Electron-beam radiolysis provides a mechanistic basis for the strong antibacterial activity observed here by enabling dose-controlled nanoparticle nucleation and growth while minimizing residual reaction by-products on the AgNP surface. These features are likely to strengthen structure–activity relationships and enhance antibacterial efficacy. Accordingly, the dose-dependent inhibition and differential responses of Gram-positive and Gram-negative bacteria indicate that the CS–AgNPs produced by this method exhibit antibacterial activity comparable to, or greater than, that of analogous systems prepared by conventional chemical or biological methods.

## 4. Materials and Methods

### 4.1. Materials

AgNO_3_ with a molecular weight of 169.87 g/mol was used as the silver precursor in this study. Chitosan, with a degree of deacetylation (%DD) of 95% and an average molecular weight of approximately 150,000 g/mol, was obtained from Seafresh Chitosan Lab Co., Ltd. (Bang Rak, Bangkok, Thailand). CH_3_COOH was purchased from RCI Labscan Limited (Pathum Wan, Bangkok, Thailand) and was used as the solvent for chitosan dissolution and pH adjustment. All solutions were prepared and stored in 20 mL glass containers. A magnetic stirrer was used for homogeneous mixing, and an analytical balance was employed for precise weighing of reagents. Electron beam irradiation was performed using a 10 MeV electron accelerator (MB20-16, MEVEX, Stittsville, ON, Canada) operated at a beam current of 10 mA, under atmospheric conditions using a conveyor-based system. The absorbed dose was controlled by adjusting conveyor speed and beam current, and samples were irradiated over a total dose range of 0–30 kGy. Dosimetry calibration was routinely performed by the irradiation facility to ensure accurate dose delivery. Irradiation was carried out at the Gems Irradiation Center, Thailand Institute of Nuclear Technology (Public Organization), Ongkharak, Nakhon Nayok, Thailand.

### 4.2. Preparation of CS–AgNPs Using an Electron Beam Irradiation

A 1 mM AgNO_3_ solution was prepared by dissolving 0.17 g of AgNO_3_ in 100 mL of distilled water. This solution was used as the silver precursor in all subsequent formulations. Chitosan solution was prepared by dissolving 2 g of chitosan powder in a 0.5% (*v*/*v*) CH_3_COOH solution, with the final volume adjusted to 1000 mL. The solution was stirred continuously using a magnetic stirrer until complete dissolution was achieved, yielding a 0.2% (*w*/*v*) stock solution. To evaluate the effects of varying chitosan concentrations, working solutions were prepared at 0.02, 0.05, 0.10, 0.50, 1.00, and 1.50% (*w*/*v*). The volumes of chitosan solution, CH_3_COOH, and distilled water were adjusted accordingly to maintain a constant total volume for each sample. The AgNO_3_ solution was then added to achieve final concentrations of 0.02, 0.04, 0.06, 0.08, and 0.10 mmol. Each silver concentration was combined with all six chitosan concentrations, resulting in a total of 30 formulations. All mixtures were shaken to ensure homogeneity. Following preparation, samples were subjected to electron beam irradiation using a linear accelerator operating at 8 MeV. Irradiation was performed at doses of 0, 1, 3, 5, 8, 10, 20, and 30 kGy to investigate the effects of radiation under controlled conditions.

The radiolytic formation of CS–AgNPs proceeds through a sequence of well-defined radiation-induced reactions. Upon electron beam irradiation, water molecules undergo radiolysis to generate reactive species, including hydrated electrons ($e_{aq}^{-}$) and hydrogen radicals (·H), which act as the primary reducing agents. These reactive species reduce Ag^+^ ions, released from AgNO_3_, to metallic silver atoms (Ag^0^), thereby initiating nucleation and subsequent nanoparticle growth. As irradiation continues, silver atoms aggregate to form stable silver nanoparticles. Simultaneously, chitosan—protonated in the presence of acetic acid—coordinates to the nanoparticle surface through its amino and hydroxyl functional groups, effectively capping and stabilizing the growing AgNPs and preventing aggregation (Equations (1)–(8)).

Dissociation of precursor

(1) $${AgNO}_{3}\left(aq\right)\to {Ag}^{+}\left(aq\right)+{NO}_{3}^{-}(aq)$$

Primary water radiolysis under electron beam

(2) $$H_{2}O\overset{e^{-}beam}{\to }e_{aq}^{-}+\cdot OH+\cdot H+H_{2}+H_{2}O_{2}$$

Reduction of silver ions to metallic silver atoms

(3) $${Ag}^{+}+e_{aq}^{-}\to {Ag}^{0}$$

(4) $${Ag}^{+}+\cdot H\to {Ag}^{0}+H^{+}$$

Nucleation and growth (cluster formation to nanoparticles)

(5) $${Ag}^{0}+{Ag}^{0}\to {Ag}_{2}^{0}$$

(6) $${Ag}_{n}^{0}+{Ag}^{0}\to {Ag}_{n+1}^{0}\to AgNP({Ag}^{0})$$

Chitosan solubilization (protonation in CH_3_COOH; stabilization)

(7) $$CS-{NH}_{2}+{CH}_{3}COOH⇌CS-{NH}_{3}^{+}+{CH}_{3}{COO}^{-}$$

Chitosan coordination/capping of nanoparticles

(8) AgNP+CS→CS−AgNP

where chitosan binds primarily via −NH_2_/−OH groups, limiting aggregation through electrostatic and steric stabilization.

### 4.3. UV–Vis Spectroscopic Analysis

The optical properties of the synthesized CS–AgNPs were characterized using a UV–visible spectrophotometer (Shimadzu UV-2500, Shimadzu Corporation, Kyoto, Japan) over a wavelength range of 300–700 nm. Measurements were performed to determine the maximum absorption wavelength (λ_max_) and the corresponding absorbance of the silver–chitosan solutions. The effects of radiation dose, AgNO_3_ concentration, and chitosan concentration on the absorbance spectra were systematically examined to assess their influence on nanoparticle formation and stability.

### 4.4. Determine the Particle Size of the CS–AgNPs Using TEM

Morphology and particle size were analyzed using a 200 kV ultra-high-resolution transmission electron microscope (JEOL-2010, JEOL Ltd., Tokyo, Japan). For TEM imaging, a drop of the CS–AgNP suspension was placed on carbon-coated copper grids, dried at ambient temperature, and examined directly. Particle size was measured from TEM micrographs using SemAfore 5.21 software (Insinööritoimisto Rimppi Oy, Ojakkala, Finland), and size distribution was analyzed using ImageJ software version 1.54p (U.S. National Institutes of Health, Bethesda, MD, USA). Samples were prepared using standard laboratory glassware and micropipettes.

### 4.5. Dynamic Light Scattering (DLS) and Zeta Potential Analysis

The hydrodynamic diameter and PDI of AgNO_3_, CS–HOAc, and CS–AgNP dispersions were measured by DLS using a ZetaPALS analyzer (Brookhaven Instruments Corporation, Nashua, NH, USA). Samples were diluted with deionized water and analyzed at 25 °C using a 633 nm laser at a scattering angle of 173°. The refractive index was set to 1.33 for water and 1.59 for AgNPs, with a dispersant viscosity of 0.8872 cP. Zeta potential was determined by electrophoretic light scattering using the same instrument and converted from electrophoretic mobility using the Smoluchowski approximation. AgNO_3_ solution was analyzed similarly; however, no defined particle size distribution was detected, consistent with its ionic nature.

### 4.6. Analyze the Morphology and Crystallinity of the CS–AgNPs Using XRD

X-ray diffractograms on powder samples were obtained using a Bruker’s X-ray Diffraction with Cu tube radiation (k = 1.54184 Å), a graphite monochromator and Lynxeye detector at 30 kV, and a current of 10 mA. The diffractometer was controlled and operated by a PC with the DIFFRAC.SUITE™ Software package with DIFFRAC.EVA V7 (Bruker AXS GmbH, Karlsruhe, Germany). Measurements were taken over an angular range of 0.99° ≤ 2θ ≤ 89.99° with a scanning step of 0.05° and a fixed counting time of 10 s. Divergence, scattered, and receiving radiation slits were 1°, 1°, and 0.2 mm, respectively [53,54]. To produce flat pellets, the CS–AgNPs were ground for two minutes using a hydraulic press of five-ton capacity. After that, the AgNPs were put in the XRD sample container for investigation with XRD patterns generated under Cu-Kα radiation (λ = 1.5406 Å) throughout a 10–90° 2θ scan range [55]. The crystalline structure of the CS–AgNPs was subsequently found by matching the diffraction peaks with the Powder Diffraction File database [56].

### 4.7. FTIR Analysis

FTIR spectroscopy was used to characterize the functional groups and chemical structure of the silver–chitosan system using an FTIR spectrometer (VERTEX 70, Bruker Daltonics GmbH & Co. KG, Bremen, Germany). Spectra were collected in attenuated total reflection (ATR) mode over the range of 400–4000 cm^−1^ with 64 scans per sample, employing a deuterated L-alanine-doped triglycine sulfate (DLATGS) detector and potassium bromide (KBr) precision windows. Prior to analysis, samples were mixed with KBr and compressed into pellets to minimize moisture interference. FTIR analysis was performed to assess the molecular structure and chemical bonding of CS–AgNPs [57].

### 4.8. Antibacterial Activity Evaluation

#### 4.8.1. Antibacterial Efficacy of CS–AgNPs Against *S. aureus* and *E. coli* via Disc Diffusion Method

The disc diffusion technique was employed to evaluate the antibacterial activity of the CS–AgNPs generated against two bacterial strains: *S. aureus* (Gram-positive) and *E. coli* (Gram-negative). The two isolates were isolated from a microbial culture collection and incubated in nutrient broth at 37 °C for the night. The bacterial suspensions were adjusted to achieve the turbidity of the 0.5 McFarland standard, which is equivalent to approximately 1.5 × 10^8^ CFU/mL. Mueller-Hinton agar (MHA) plates were prepared and inoculated by equitably disseminating a bacterial suspension across the entire surface using sterile cotton swabs. Sterile Whatman No. 1 filter paper discs (6 mm in diameter) were impregnated with 10–20 μL of CS-AgNPs suspensions at varying concentrations and air-dried under aseptic conditions. Next, the discs were carefully set on the surface of the infected agar plates. A positive control of commercial Gentamicin antibiotic discs was employed for comparison, while a negative control of discs containing sterile water was employed. The dishes were incubated at 37 °C for 24 h. The antibacterial activity of the discs was assessed by measuring the width of the zone of inhibition encircling each disc after incubation using a digital caliper.

#### 4.8.2. Assessment of the Antibacterial Activity of CS–AgNPs Against *S. aureus* and *E. coli* Through MIC and MBC Analysis

**Figure 12:**
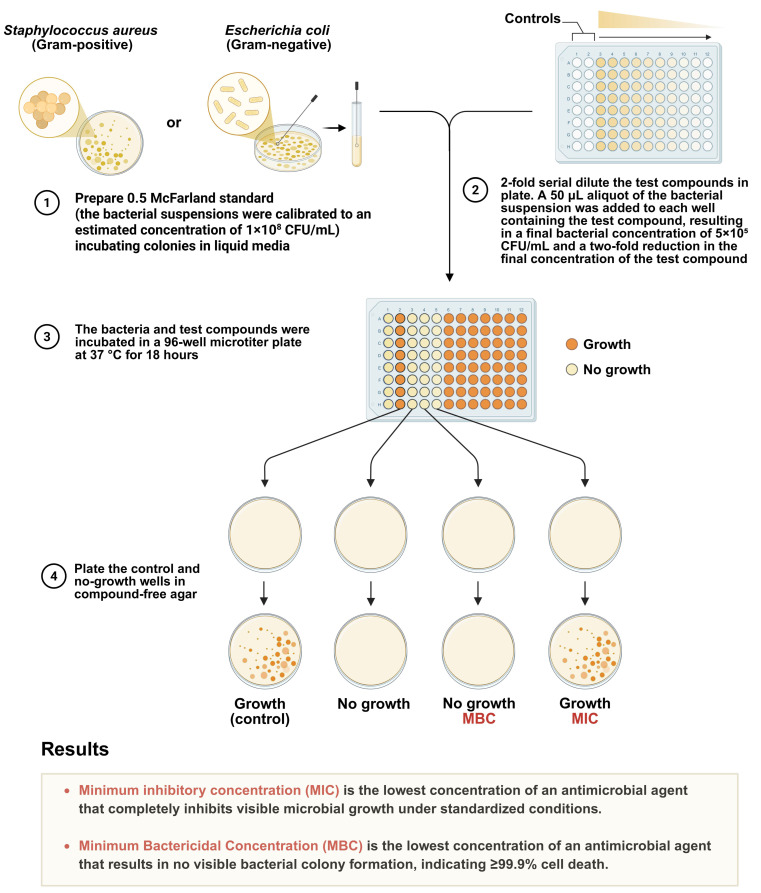
Schematic illustration of the procedure used to determine the MIC and MBC of CS–AgNPs against *S. aureus* (Gram-positive) and *E. coli* (Gram-negative). (**1**) Bacterial suspensions were adjusted to a 0.5 McFarland standard (≈1 × 10^8^ CFU/mL). (**2**) The test compounds were serially diluted two-fold in a 96-well microtiter plate, and 50 µL of bacterial inoculum (≈5 × 10^5^ CFU/mL) was added to each well. (**3**) Plates were incubated at 37 °C for 18 h, and bacterial growth was visually assessed. (**4**) Wells showing no visible growth were subcultured onto compound-free agar to determine bactericidal activity. The lowest concentration preventing visible growth was recorded as the MIC, while the lowest concentration resulting in no bacterial colony formation (≥99.9% killing) was designated as the MBC.

Two bacterial strains were used for antibacterial testing: *S. aureus* and *E. coli*. The strains were acquired from a recognized microbial culture collection and preserved on MHA. Before each experiment, bacteria were cultivated in Mueller-Hinton Broth (MHB) and incubated overnight at 37 °C to guarantee active proliferation. The bacterial suspensions were calibrated to an estimated concentration of 1 × 10^6^ CFU/mL for the MIC and MBC experiments (Figure 12).

The MIC and MBC of CS–AgNPs were ascertained utilizing the broth microdilution technique, in accordance with the standards set by the Clinical and Laboratory Standards Institute (CLSI). Two-fold serial dilutions of CS–AgNPs were formulated in sterile MHB to achieve final concentrations between 1 and 512 μg/mL. Each well of a sterile 96-well microtiter plate was filled with 100 μL of the diluted nanoparticle solution, followed by the addition of 100 μL of bacterial suspension, yielding a final volume of 200 μL per well. Each plate featured positive controls (bacteria with MHB only), negative controls (MHB without bacteria), and DI water controls (to evaluate the effect without silver).

Post-inoculation, the plates were incubated at 37 °C for 18–24 h under static conditions. The MIC was determined as the minimal concentration of CS–AgNPs that entirely prevented observable bacterial proliferation, evaluated by the transparency of the broth. To ascertain the MBC, 10 μL aliquots were extracted from wells exhibiting no discernible growth and inoculated into fresh MHA plates. The plates were subsequently incubated for a further 24 h at 37 °C. The MBC was determined to be the minimal concentration of CS–AgNPs that produced no detectable colony formation, signifying at least 99.9% bacterial lethality [58,59,60,61,62].

#### 4.8.3. Biofilm Inhibition Assay

Biofilm inhibition was evaluated using a crystal violet microplate assay. *S. aureus* and *E. coli* were cultured overnight in tryptic soy broth (TSB) at 37 °C. The bacterial suspension was adjusted to approximately 1 × 10^6^ CFU/mL. Aliquots (100 µL) of bacterial suspension were added to sterile 96-well flat-bottom polystyrene microplates, followed by 100 µL of CS–AgNPs diluted in TSB to obtain final concentrations corresponding to 0.5 × MIC, 1 × MIC, 2 × MIC, and 4 × MIC. Wells containing bacteria without treatment served as growth controls, while media-only wells were used as blanks. Plates were incubated statically at 37 °C for 24 h to allow biofilm formation in the presence of the test material. After incubation, planktonic cells were carefully removed, and wells were gently washed three times with PBS to eliminate non-adherent bacteria. Biofilms were stained with 0.1% (*w*/*v*) crystal violet solution for 15 min at room temperature. Excess stain was removed by washing with distilled water. The bound dye was solubilized using 200 µL of 95% ethanol, and absorbance was measured at 570 nm using a microplate reader.

Biofilm inhibition (%) was calculated as:

(9) $$Inhibition\;\left(\%\right)=\;\frac{{OD}_{control}-{OD}_{treated}}{{OD}_{control}}\;\times 100$$

#### 4.8.4. Biofilm Eradication Assay (Mature Biofilm Model)

To evaluate disruption of pre-formed biofilms, bacterial suspensions (1 × 10^6^ CFU/mL) were seeded in 96-well plates and incubated at 37 °C for 24 h to allow mature biofilm formation. After removal of planktonic cells and washing with PBS, fresh TSB containing CS–AgNPs (0.5 × MIC, 1 × MIC, 2 × MIC, and 4 × MIC) was added. Plates were incubated for an additional 24 h at 37 °C. Biofilm biomass was quantified using crystal violet staining as described above. Biofilm eradication (%) was calculated relative to untreated mature biofilm controls.

### 4.9. In Vitro Biocompatibility Evaluation

#### 4.9.1. In Vitro Cytocompatibility Evaluation Using HaCaT Cells

Human immortalized keratinocyte cells (HaCaT) were cultured in Dulbecco’s Modified Eagle Medium (DMEM) supplemented with 10% fetal bovine serum and maintained at 37 °C in a humidified atmosphere containing 5% CO_2_. For cytotoxicity assessment, cells were seeded in 96-well plates at a density of 1 × 10^4^ cells per well in 100 μL of supplemented DMEM and incubated at 37 °C in a humidified atmosphere with 5% CO_2_ for 4 h to allow cell attachment. Subsequently, non-adherent cells were removed by gently washing with fresh DMEM. CS–AgNPs, AgNO_3_, and chitosan solutions were prepared in supplemented DMEM to obtain final concentrations of 5, 25, 50, 100, 250, 500, 1000, and 2000 μg/mL. The cells were exposed to the respective treatments and incubated for 48 h under standard culture conditions (37 °C, 5% CO_2_). Untreated cells served as the negative control (100% viability), while cells treated with 10% (*v*/*v*) dimethyl sulfoxide (DMSO) were used as the positive control. To exclude potential interference of silver nanoparticles with the MTT assay (e.g., optical absorbance overlap or direct reduction of MTT), nanoparticle-only control wells (without cells) were included at each tested concentration and processed identically. The absorbance values obtained from these wells were subtracted from the corresponding sample readings prior to viability calculation.

Following treatment, 10 μL of MTT solution (5 mg/mL in phosphate-buffered saline, PBS) was added to each well, and the plates were further incubated for 4 h to allow formazan crystal formation. The supernatant was carefully aspirated to minimize nanoparticle carryover, and the resulting formazan crystals were dissolved by adding 100 μL of DMSO to each well with gentle shaking to ensure complete solubilization. Absorbance was measured at 550 nm using a microplate reader. Cell viability (%) was calculated relative to the untreated control group after background correction.

#### 4.9.2. In Vitro Hemolysis Assay and Blood Compatibility Evaluation

Human whole blood was collected in heparinized tubes to prevent coagulation. Plasma was removed by centrifugation, and the red blood cells (RBCs) were washed three times with PBS to remove residual plasma proteins. The purified RBCs were then resuspended in PBS at a final concentration of approximately 8 × 10^9^ cells/mL. The RBC suspensions were incubated with CS-AgNPs at concentrations of 50 and 500 μg/mL. RBCs treated with 0.1% Triton X-100 served as the positive control (100% hemolysis), while RBCs suspended in 1 × PBS served as the negative control (0% hemolysis). All samples were incubated at 37 °C for 12 and 24 h under gentle mixing. Following incubation, samples were centrifuged at 500× *g* for 5 min to pellet intact RBCs. The supernatants were carefully collected, and hemoglobin release was quantified by measuring absorbance at 562 nm using a microplate reader. Hemolysis percentage was calculated relative to the positive and negative controls.

### 4.10. Statistical Analysis

All experiments were performed in triplicate (*n* = 3) and independently repeated at least three times unless otherwise stated. Data are presented as mean ± standard deviation (SD). For comparisons involving more than two groups (e.g., concentration-dependent biofilm inhibition, eradication assays, cytotoxicity dose–response analysis, and hemolysis evaluation), statistical significance was assessed using one-way analysis of variance (ANOVA) followed by Tukey’s post hoc multiple comparison test. For direct pairwise comparisons between two bacterial species (*S. aureus* vs. *E. coli*) at identical treatment conditions (e.g., MIC, MBC, and specific concentration points in biofilm assays), an unpaired two-tailed Student’s *t*-test was applied. A *p*-value < 0.05 was considered statistically significant. Statistical analyses were performed using GraphPad Prism (version 10.6, GraphPad Software, San Diego, CA, USA).

## 5. Conclusions

This study establishes electron-beam irradiation as a powerful, clean, and scalable platform for the synthesis of chitosan–silver nanocomposites with strong biomedical relevance. The resulting CS–AgNPs exhibited phase-pure, highly crystalline FCC silver uniformly integrated within the chitosan matrix, with XRD confirming enhanced crystallinity and FTIR verifying strong Ag–polymer coordination through amino, hydroxyl, and carbonyl groups. These structural features directly support nanoparticle stability, dispersion, and functional integrity. The nanocomposites also showed clear concentration-dependent antibacterial activity against both *S. aureus* and *E. coli*, with greater effectiveness against the Gram-positive strain, highlighting the combined contribution of Ag^+^ release, reactive crystal surfaces, and chitosan-assisted biointeractions. Importantly, this radiolytic strategy achieved tunable physicochemical properties and high phase purity without chemical reducing agents or stabilizers, emphasizing its environmental advantage over conventional synthesis methods. Collectively, these findings position electron-beam radiolysis as a highly promising route for the development of next-generation antimicrobial nanomaterials and provide a strong foundation for future translation into wound care, biomedical coatings, and drug delivery systems.

## Figures and Tables

**Table 1:** Hydrodynamic diameter, PDI, and zeta potential values of AgNO_3_, CS–HOAc, and CS–AgNP dispersions measured by DLS and electrophoretic mobility analysis. Data are presented as mean ± standard deviation (*n* = 3).

Sample	Size (nm)	PDI	Zeta Potential (mV)	Interpretation
AgNO_3_	No defined peak	—	−8 ± 2	Ionic species, no colloidal stability
CS–HOAc	220 ± 30	0.45 ± 0.13	42 ± 3	Protonated polymer chains
CS–AgNPs	55 ± 6	0.26 ± 0.07	34 ± 2	Stable coated nanoparticles

## Data Availability

The original contributions presented in this study are included in the article material. Further inquiries can be directed to the corresponding author.

## Supplementary Materials

The following supporting information can be downloaded at: https://www.mdpi.com/article/10.3390/ijms27062569/s1.
