# Supplementary material for: Radiolytic Synthesis of Chitosan-Stabilized Silver Nanoparticles via Electron Beam Irradiation for Enhanced Antibacterial Activity Against Staphylococcus aureus and Escherichia coli
Source: Int J Mol Sci. 2026 Mar 11;27(6):2569. doi: 10.3390/ijms27062569 (PMC13026173; doi:10.3390/ijms27062569)
Supplement: Supplementary file 1 [file ijms-27-02569-s001.zip › ijms-4158557-supplementary.pdf]

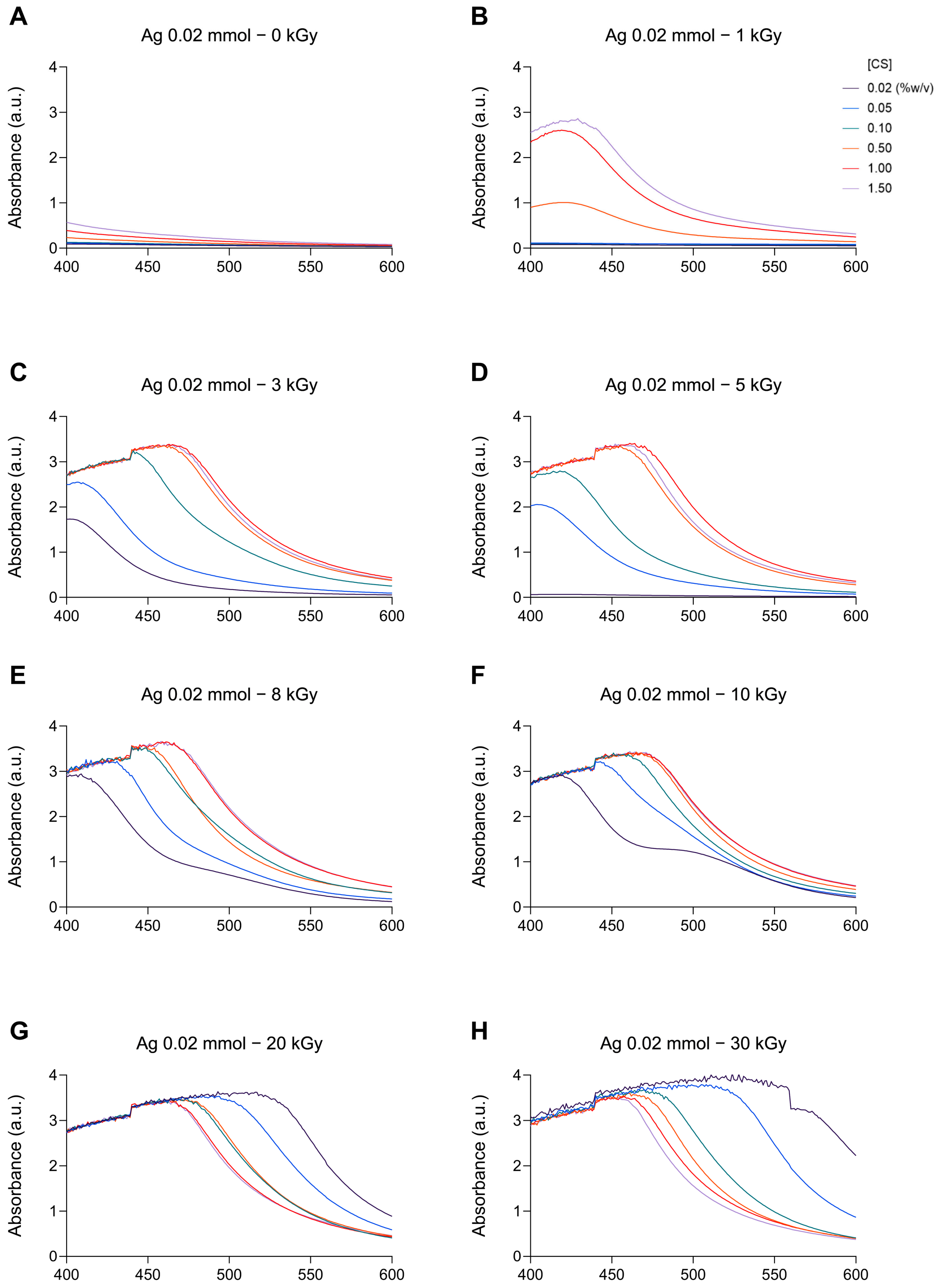

Figure S1. UV–Vis absorption spectra of CS–AgNPs prepared using 0.02 mmol $AgNO_3$ and chitosan concentrations of 0.02, 0.05, 0.10, 0.50, 1.00, and 1.50% (w/v) under electron beam irradiation at doses of (**A**) 0, (**B**) 1, (**C**) 3, (**D**) 5, (**E**) 8, (**F**) 10, (**G**) 20, and (**H**) 30 kGy.

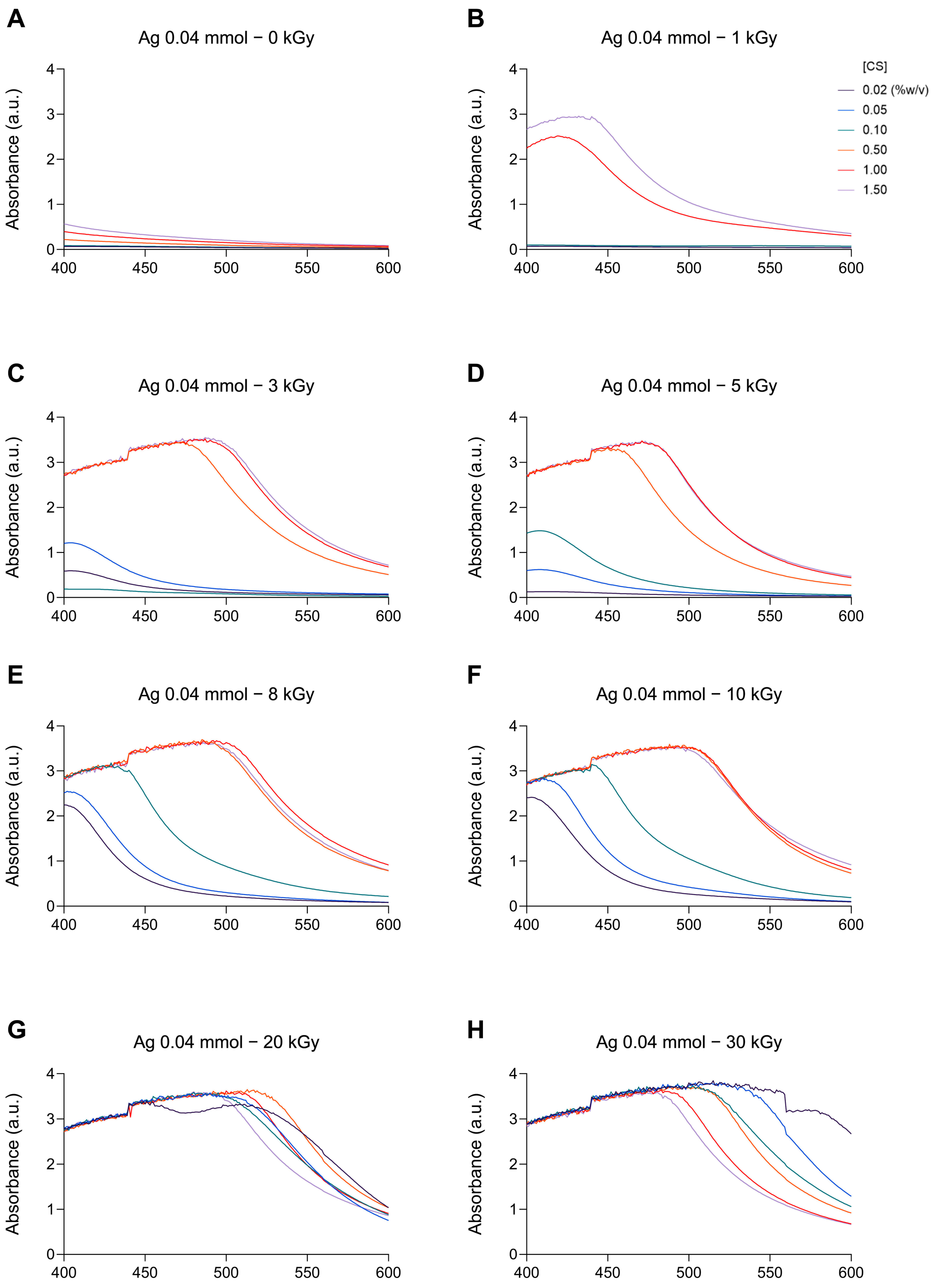

Figure S2. UV–Vis absorption spectra of CS–AgNPs prepared using 0.04 mmol $AgNO_3$ and chitosan concentrations of 0.02, 0.05, 0.10, 0.50, 1.00, and 1.50% (w/v) under electron beam irradiation at doses of (**A**) 0, (**B**) 1, (**C**) 3, (**D**) 5, (**E**) 8, (**F**) 10, (**G**) 20, and (**H**) 30 kGy.

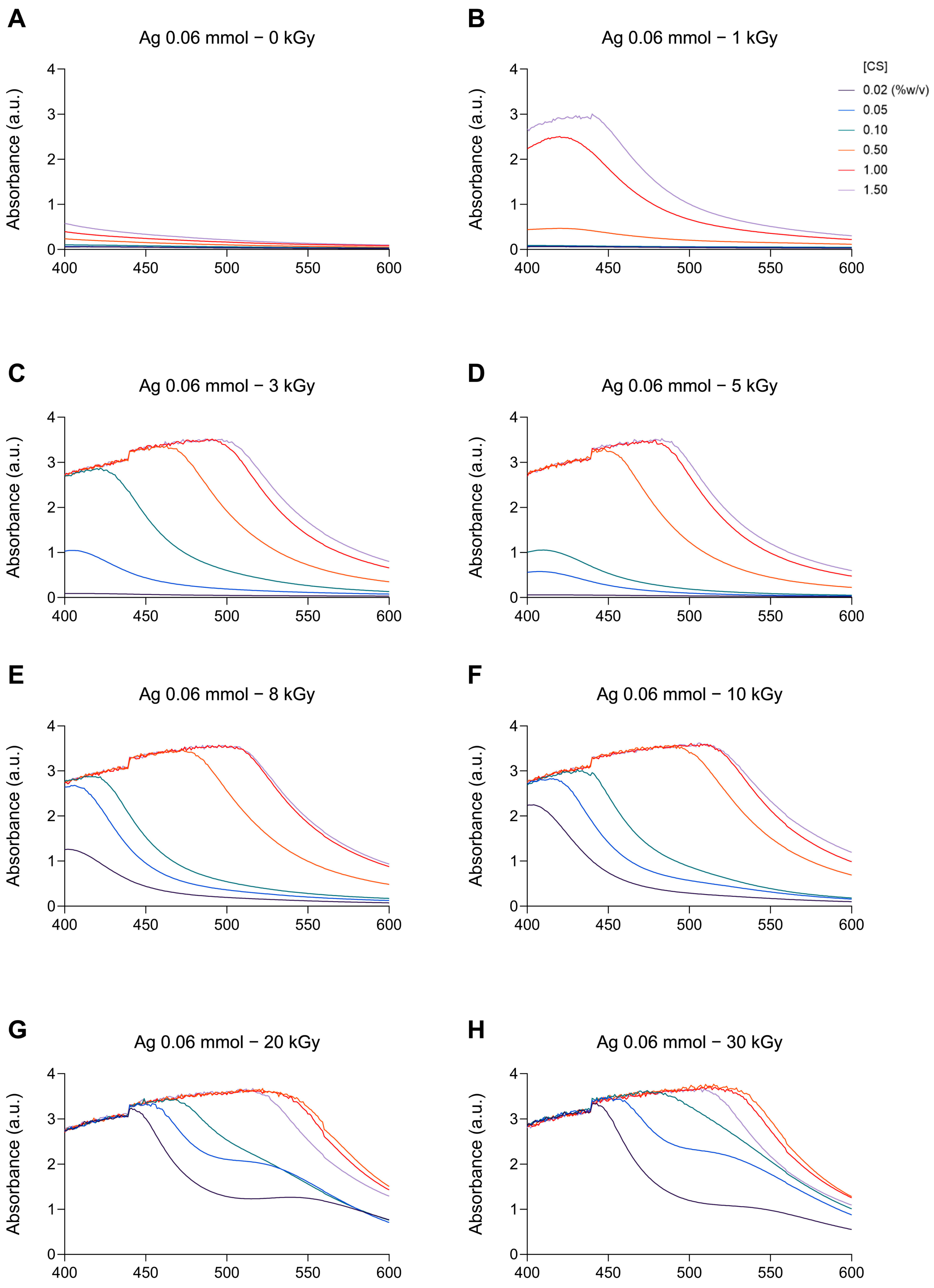

Figure S3. UV–Vis absorption spectra of CS–AgNPs prepared using 0.06 mmol $AgNO_3$ and chitosan concentrations of 0.02, 0.05, 0.10, 0.50, 1.00, and 1.50% (w/v) under electron beam irradiation at doses of (**A**) 0, (**B**) 1, (**C**) 3, (**D**) 5, (**E**) 8, (**F**) 10, (**G**) 20, and (**H**) 30 kGy.

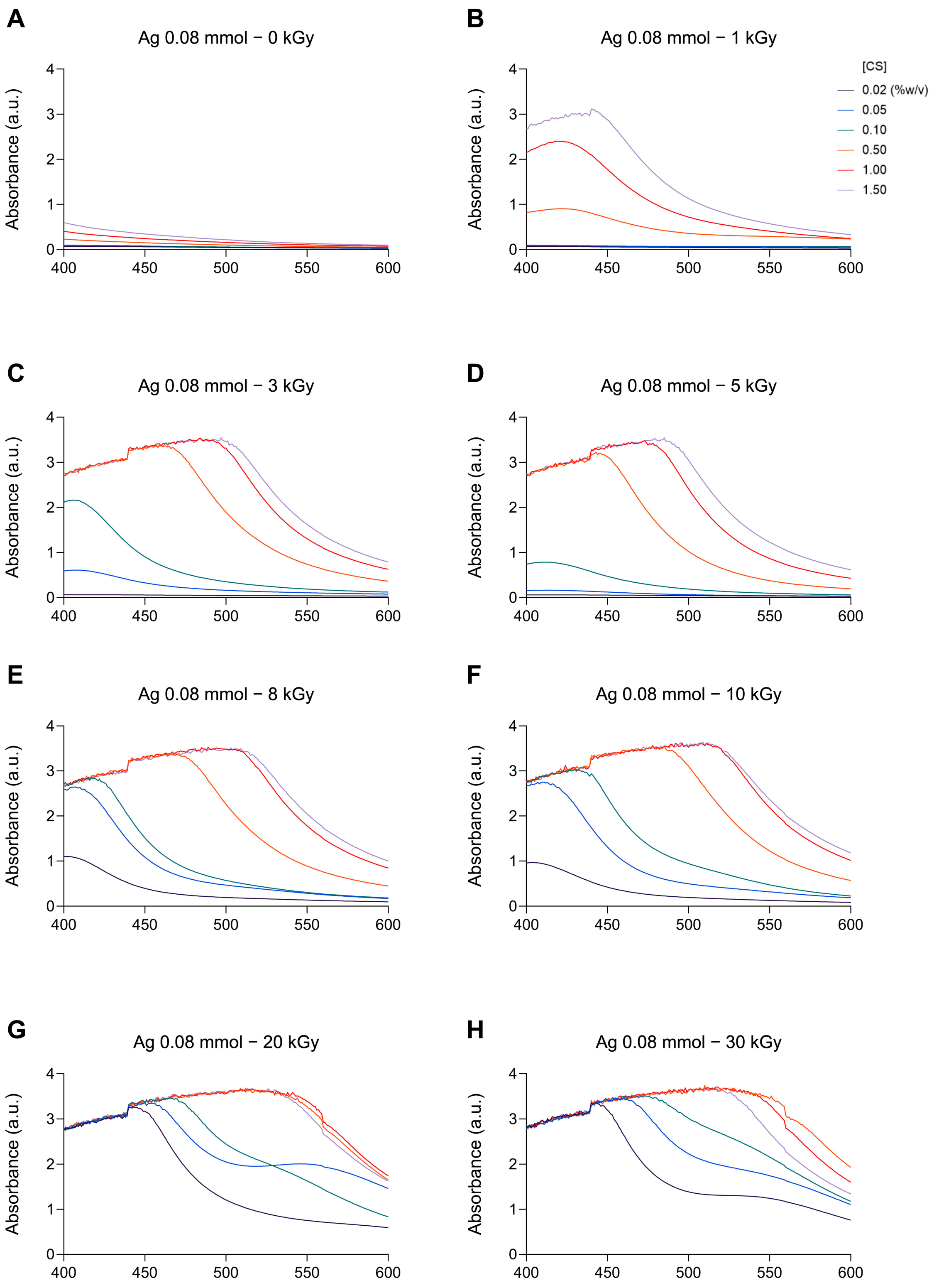

Figure S4. UV–Vis absorption spectra of CS–AgNPs prepared using 0.08 mmol $AgNO_3$ and chitosan concentrations of 0.02, 0.05, 0.10, 0.50, 1.00, and 1.50% (w/v) under electron beam irradiation at doses of (**A**) 0, (**B**) 1, (**C**) 3, (**D**) 5, (**E**) 8, (**F**) 10, (**G**) 20, and (**H**) 30 kGy.

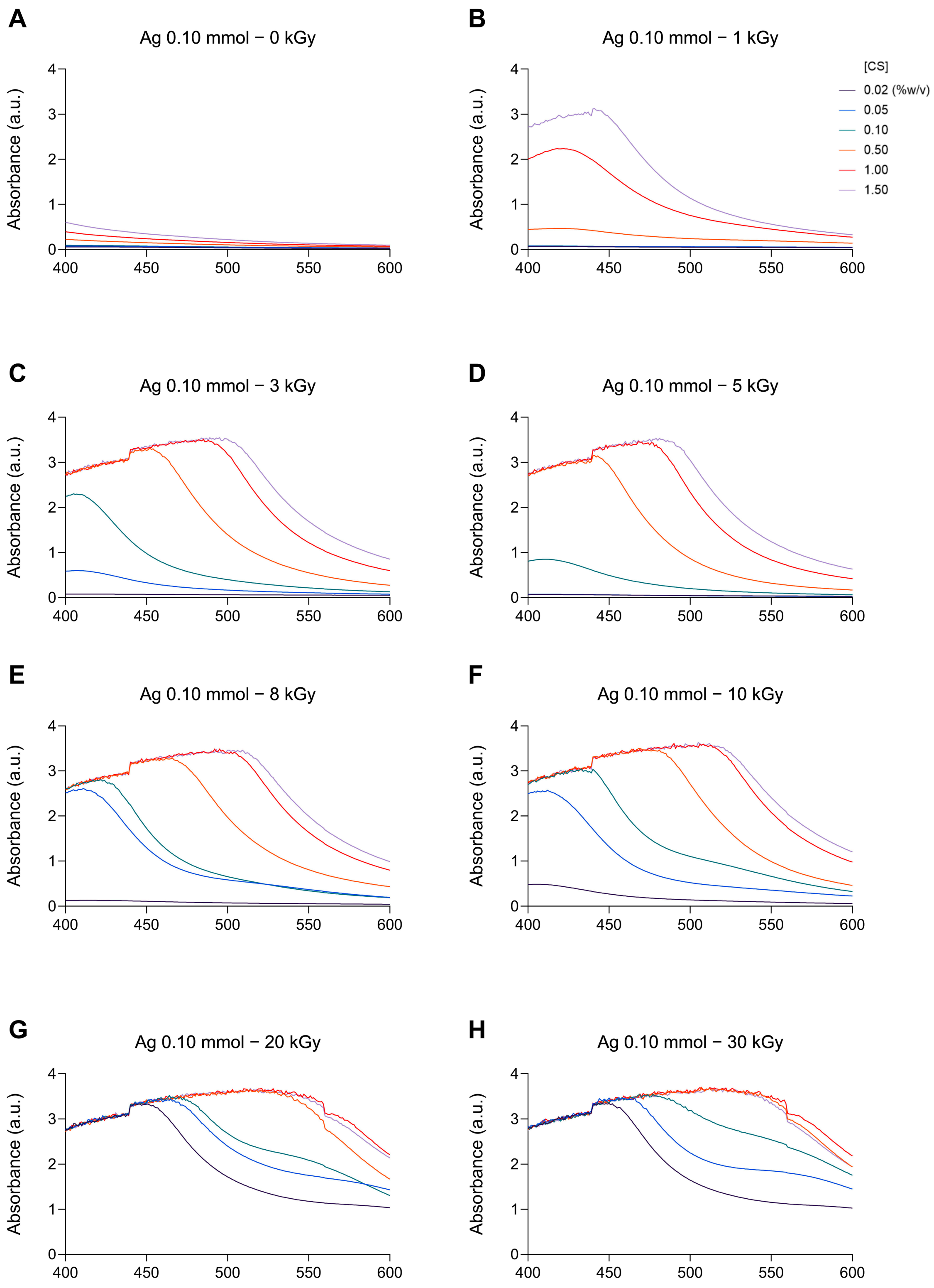

Figure S5. UV–Vis absorption spectra of CS–AgNPs prepared using 0.10 mmol $AgNO_3$ and chitosan concentrations of 0.02, 0.05, 0.10, 0.50, 1.00, and 1.50% (w/v) under electron beam irradiation at doses of (**A**) 0, (**B**) 1, (**C**) 3, (**D**) 5, (**E**) 8, (**F**) 10, (**G**) 20, and (**H**) 30 kGy.
